# Heat-Shock Proteins in Leukemia and Lymphoma: Multitargets for Innovative Therapeutic Approaches

**DOI:** 10.3390/cancers15030984

**Published:** 2023-02-03

**Authors:** Vincent Cabaud-Gibouin, Manon Durand, Ronan Quéré, François Girodon, Carmen Garrido, Gaëtan Jego

**Affiliations:** 1 INSERM, UMR1231, Université de Bourgogne, 21078 Dijon, France; 2 Service d’Hématologie Biologique, Hôpital Universitaire François Mitterrand, 21000 Dijon, France; 3 Centre Georges François Leclerc, 21000 Dijon, France

**Keywords:** heat-shock protein, leukemia, lymphoma, targeted therapy

## Abstract

**Simple Summary:**

Heat-shock proteins (HSPs) are molecular chaperones overexpressed in tumor cells and are necessary for their survival. In leukemia and lymphoma, HSPs have been reported to have unique cytoprotective effects on different cell death and growth pathways. In this review, we describe the implication of HSPs in those pathways in hematological malignancies and discuss the pertinence of detecting and targeting them for future innovative treatment strategies.

**Abstract:**

Heat-shock proteins (HSPs) are powerful chaperones that provide support for cellular functions under stress conditions but also for the homeostasis of basic cellular machinery. All cancer cells strongly rely on HSPs, as they must continuously adapt to internal but also microenvironmental stresses to survive. In solid tumors, HSPs have been described as helping to correct the folding of misfolded proteins, sustain oncogenic pathways, and prevent apoptosis. Leukemias and lymphomas also overexpress HSPs, which are frequently associated with resistance to therapy. HSPs have therefore been proposed as new therapeutic targets. Given the specific biology of hematological malignancies, it is essential to revise their role in this field, providing a more adaptable and comprehensive picture that would help design future clinical trials. To that end, this review will describe the different pathways and functions regulated by HSP27, HSP70, HSP90, and, not least, HSP110 in leukemias and lymphomas.

## 1. Introduction

Leukemias and lymphomas represent 6.5% of cancers worldwide [1]. They emerge from a wide array of cells at different stages of hematopoietic maturation, from hematopoietic stem cells and myeloid and lymphoid progenitors to mature B and T cells. Treatment breakthroughs have been made in recent years thanks to the comprehensive identification of survival pathways and oncogenes, leading to targeted therapies such as kinase inhibitors or anti-apoptotic protein inhibitors [2,3]. At the same time, recent progress in high-throughput molecular analysis combined with bioinformatic power has revealed a more diverse and complex picture of cancer sub-populations than expected, challenging the too simplistic “one treatment fits all” [4]. Furthermore, natural mechanisms of cell death resistance and escape from therapy occur in many patients either from the start of the treatment or after several cycles of chemotherapy, pushing for more specific and innovative drug use in the clinic. Therefore, cancer initiation, development, and cell death resistance are still intense fields of research to identify new therapeutic targets that could foster the development of new treatments.

HSPs have been involved in many cellular processes as they are important housekeeping proteins. Their chaperone function is observed in every cell compartment and is intrinsically associated with protein translation, migration, localization, stability, and degradation. Traditionally, they have been classified by their molecular weight, with the most studied members in cancer being HSP90, HSP70, HSP27, and, more recently, HSP110. Cell stress is a powerful initiator and booster of HSP expression as it leads to multiple protein structural alterations, such as aggregation, unfitted conformations, and accelerated protein synthesis. Because tumor cells must rewire their metabolism in permanence, they are like highly stressed cells and therefore rely on the strong expression of HSPs for their survival. Consequently, they become more resistant to cell death mechanisms, either of natural origin or therapeutically induced. HSP expression profiles in newly diagnosed patients compared to healthy controls in various hematological malignancies reveal a frequent but heterogeneous expression. In acute myeloid leukemia (AML), HSP70 was strongly expressed in 58% of patients, compared to 26% for HSP60 [5]. Despite this variability of expression, the global HSP family proteome provides widespread protection to tumor cells, as the overall survival of AML patients was inversely correlated to HSP expression. Moreover, when other prognosis factors were considered, HSP expression always an additive pejorative value for the patients’ outcomes [5].

Similarly, in chronic lymphocytic leukemia (CLL) and chronic myeloid leukemia (CML), HSP70 is significantly more expressed in patients than in healthy controls. High levels of HSP70 correlate with resistance to tyrosine kinase inhibitors [6,7]. In lymphoma, a high expression of the mitochondrial HSP70 (mortalin) correlates with treatment resistance and poor survival of patients [8]. As HSP expression is highly modulated by environmental aggressions, HSPs further accumulate in cancer cells after anti-cancer treatments through the induction/activation of the HSPs’ transcription factor HSF1. In CLL, increased HSP70 and HSF1 were observed in response to Ibrutinib treatment and signaled a failure of clinical improvement [9]. Conversely, only responders show a decrease in HSP70 expression [7]. HSP27 has been associated with clinical responses as it is highly expressed in pediatric acute leukemia and provides resistance to chemotherapy [10]. HSPs are also secreted, free or with nanovesicules. Some of them, such as HSP70, have a membrane location and can be found both in the cancer microenvironment and in the blood [11]. High HSP70 levels have been detected at the cell surface and in the serum in AML and correlate with limited survival [12,13]. Extracellular HSPs, also described in many solid tumors, can have an immunosuppressive function mostly through macrophage polarization and myeloid suppressive cell activation [14]. The importance of the immune microenvironment in leukemia and lymphoma [15] and the capacity of HSPs to diffuse largely through the body as free proteins or embedded in extracellular vesicles provide solid grounds for studies of their extracellular functions. The most abundant HSP, HSP90, also shows elevated levels in various types of leukemias and lymphomas and could serve as a prognostic marker. In addition, its elevated expression is necessary for the survival and propagation of those cancer cells [16,17,18,19,20,21]. Finally, the expression of the high-molecular-weight HSP110, a long-forgotten chaperone, has recently been revealed to correlate with the aggressiveness of non-Hodgkin lymphoma (NHL) [22,23,24].

HSPs are therefore strongly associated with hematological malignancy aggressiveness and responses to therapies. In this review, we will present the most recent advances in understanding the different HSP functions in this specific family of tumors, ranging from their BCR signaling pathway to their metabolic regulation.

## 2. HSP Families’ Overview

### 2.1. Heat-Shock Factor

In humans, there are six members forming the HSF family: HSF1, HSF2, HSF4, HSF5, HSFX, and HSFY. HSF proteins have specific and overlapping roles. HSF1 is the most studied member of the family and is known to be involved in the expression of HSPs during a response to different types of stresses. However, it is now well established that HSFs are involved in the regulation of a broader array of genes that are not related to HSPs in normal and pathological hematopoiesis [25]. HSF1 is also involved in immune responses and aging. Together with HSF2, both transcription factors are involved in the development of gametes (sperm and oocytes) and the nervous system. HSF4 has been described to cooperate with HSF1 and is involved in the development and homeostasis of the lens, the survival of the lens cells, and the genesis of neurons [26]. It is also expressed in the brain, muscles, heart, and pancreas. HSF5 is one of the newest members of the family, and although it is expressed in humans, its roles are better understood in animals: in the zebrafish, HSF5 loss of function leads to male infertility, and in mice, HSF5 is known to be involved in spermatogenesis. HSFX is localized on the X chromosome, but its roles are unknown. Finally, HSFY, located on the Y chromosome, has been described as being involved in spermatogenesis, with its deletion causing infertility [27].

HSPs are transcriptionally regulated by two members of the heat shock factor family, HSF1 and HSF2. Various stresses can activate HSF1 and thereby induce HSP expression: an increase in the cytosolic levels of misfolded proteins, a rise in temperature, or an alteration in the cellular pH or redox state. Under normal conditions, HSF1 is restrained in the cytosol by HSP90 or HSP70, and with one of the cell stresses quoted above, HSF1 will dissociate from those HSPs [28]. This will allow HSF1 to phosphorylate and trimerize, and so it will enter the nucleus, binding the HSE sequences and permitting the expression of HSP genes [29]. In general, HSF1 recognizes and binds the heat-shock element (HSE) sequence (nTTCnnGAAnnTTCn) in various regions of HSP genes upon activating signals and promotes their transcription. HSF1 is involved in the proteostasis of the earliest stage of blood cell development. Notably, HSF1 ensures protein synthesis and proteostasis in hematopoietic stem cells [30]. Since HSF1 is a central regulator of stress responses, various studies have demonstrated its implication in cancer development, showing it to be an important target in cancer therapy. In AML, the inhibition of HSF1 with the chemical compound DTHIB decreases the growth and engraftment of AML cells [31]. It has also been shown that the inhibition of the translational factor eIF4a inactivates HSF1 and thereby has anti-leukemic effects both in vitro and in vivo [32]. In diffuse large B-cell lymphomas (DLBCL), HSF1 mRNA is upregulated compared with regular B-cells, which can be one of the reasons for the strong expression of a wide variety of HSPs in this type of cancer [33]. Cooperating with STAT3, HSF1 is able to maintain the cancerous phenotype of liver cancer cells [34], and the inhibition of HSF1 in pancreatic cancer decreases their stemness and sensitizes them to gemcitabine [35]. HSF1 knock-down in prostate cancer cell lines sensitizes them to chemotherapy [36]. Recently, it has been shown that HSF2, in an interplay role with HSF1, can also induce the expression of HSPs [37]. HSF2 does not have a significant role in the heat-shock response, but it is able to regulate a common set of genes with HSF1, and among them are the HSP genes. Using HSF1 and/or HSF2 knockout cell lines, dysregulated responses to nutrient stress could be observed, as well as a reduction in tumor progression, making HSF2 a critical co-factor of HSF1 [38]. HSF2 is involved in various types of carcinomas. Notably, HSF2 is known to be constitutively active in embryonic carcinoma cells, leading to the overexpression of HSPs. It is also implicated in the development of breast, lung, thyroid, oesophageal, and prostate cancers [39].

### 2.2. HSP110 Family

The HSP family with the highest molecular weight is composed of four members: HSP110 (HSP105, NY-CO-25, or HSPH1), APG2 (HSPA4 or HSPH2), APG1 (HSPA4L, Osp94, or HSPH3), and GRP170 (ORP150, HYOU1, or HSPH4). The roles of APG1 and APG2 are not well known. APG1 is involved in testis homeostasis, and APG2 has roles in spermatogenesis and is also expressed in the brain. GRP170 is an endoplasmic reticulum (ER) chaperon induced by glucose deprivation [40]. HSP110 is coded by the gene HSPH1 and was first described as a cochaperone of HSP70 and HSC70. More specifically, it was characterized as a nucleotide exchange factor (NEF) for those HSPs, carrying ATP and so enhancing their chaperoning function [41,42]. HSP110 is known to recognize and bind denatured or misfolded proteins for other HSPs, such as HSP70, to convert them into fully functional proteins [42]. Other HSPs, such as HSP70 or HSC70, are able to perform this function, but HSP110 is four times more efficient [40]. Therefore, it was thought that HSP110 was dependent on HSP70/HSC70 for its refolding role, but it has been discovered that HSP110, with the help of HSP40, can unfold and refold misfolded proteins on its own [42]. Together with HSP70 and HSP40, the HSP110-HSP70-HSP40 complex is an important chaperone network that disaggregates and refolds aggregates of denatured proteins [40].

### 2.3. HSP90 Family

HSP90 is probably the best-known family of HSPs involved in hematopoietic malignancies. Its members are well represented in cells, since they constitute between 1% and 2% of whole cellular proteins in mammals [43]. HSP90 family members are spread into different cell compartments: HSP90α A1 (also called HSPC1, HSP90A, HSP90AA1, or HSP86), HSP90α A2 (HSPC2 or HSP90AA2), and HSP90β (HSPC3, HSP90B, HSP90AB1, or HSP84) are cytosolic, while Grp94 (called HSPC4, GRP94, gp96, or HSP90B1) is restrained to the endoplasmic reticulum but can also be in the cell membrane. Finally, the last member, TRAP1 (or HSPC5, HSP90L, or HSP75), is mainly located in the mitochondria. More than 200 cellular proteins have been described as HSP90 client proteins. From proximal molecules of the BCR to transcription factors within the nucleus, HSP90 is a systematic organizer of protein transcription, translation, and activation. Abundant literature illustrates this “hub” function of HSP90 within several signaling pathways. From the cell surface, where tonic or chronic BCR stimulation occurs, HSP90 is present and interacts with BCR components to sustain signals.

### 2.4. HSP70 Family

The HSP70 family is, along with the HSP90 family, the most studied group of chaperones. There are thirteen members of the HSP70 family, all coded by the HSPA genes. Five of them are particularly studied: HSP70 (also called HSPA1 or HSP72) and HSPA6 (also called HSPB’), which are stress-induced, but also three other constitutively expressed HSP70 members, which are HSC70 (also called HSPA8 or HSP73), mortalin (also called HSPA9), and GRP78 (also called HSPA5 or BiP) [44]. HSP70s are involved in multiple cellular functions, such as the folding of unfolded or misfolded proteins, the maintenance of protein homeostasis, the transport of proteins to different subcellular fractions, and cell survival after stress [44,45]. With the help of its cochaperones, HSP40 and HSP110, HSP70 is able to desegregate and fold denatured proteins and aggregates [40]. The HSP70 family of chaperones is known for its diverse roles in cancer [44]. When it comes to lymphoid malignancies, four of these HSPs are described in the literature: HSP70 (or HSP72), HSC70, Grp78, and mortalin.

### 2.5. Small HSPs Family

The small HSP family has ten members. The best-known is HSP27 (HSPB1, HSP25, or HSP28), which is cytoplasmic but can translocate to the nucleus under stress, and it has been reported to be involved in cancer development. MKBP (HSPB2) is mainly expressed in the heart and muscles, helping with their structure and functions. HSPL27 (HSPB3, DHMN2C, or HMN2C) is also an HSP involved in muscle functions and is specific to this type of tissue. αA-crystallin (HSPB4, CRYAA, CRYA1, or CTRCT9) is principally expressed in the eye and most specifically in the lens. Unlike most chaperones, it does not refold proteins but holds them, maintaining the solubility of aggregates to maintain cell survival. The αB-crystallin (HSPB5, CRYAB, CRYA2, or MFM2) is a ubiquitous chaperone involved in the development of neurological diseases, certain myopathies, and cancers. HSP20 (HSPB6 or HEL55), like HSPB2 and HSPB3, is involved in muscle and heart functions. HSPB7 (or cvHSP) is known to be involved in heart failures and is suspected of acting as a tumor suppressor, associated with renal carcinomas. HSP22 (HSPB8, H11, HMN2, or E2IG1) has an estrogen-dependent expression in estrogen receptor-positive breast cancer cells and in some neuromuscular diseases. HSPB9 (or CT51) is the least well-known member of the family, with a cytoplasmic and nuclear location in cells. Finally, ODF1 (or HSPB10) is a chaperone involved in the cytoskeleton structures of sperm tails. In general, small HSPs have been shown to have roles in cell proliferation, survival, and the progression of cancer cells. Notably, HSPB1 is involved in breast cancer cell lines proliferation [46], and HSPB5 is associated with breast carcinoma progression [47], making HSPB5 a biomarker for the diagnosis of breast cancers [48]. High levels of HSPB5 are associated with low survival rates in hepatocellular, lung, and prostatic carcinomas [49,50,51].

## 3. HSPs as Chaperons of Cell Signaling Proteins

### 3.1. Chaperoning the B-Cell Receptor and the T-Cell-Receptor

**Figure 1:**
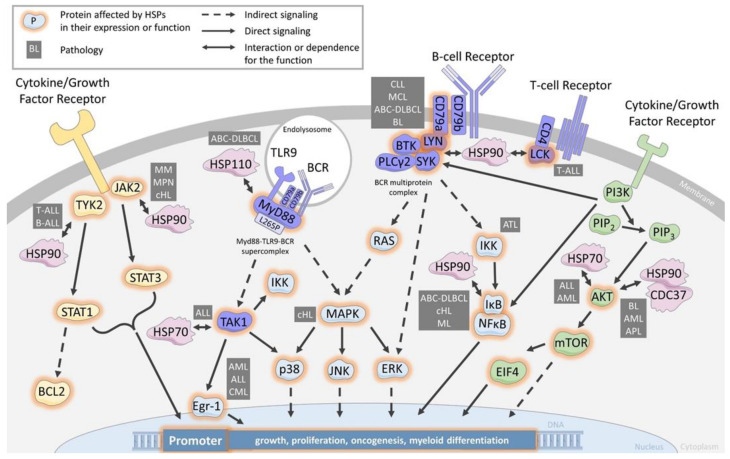
HSP are chaperones of cell signaling components in the BCR/TCR, JAK/STAT and PI3K/AKT pathways. Schematic of a leukemia/lymphoma cell showing the main actors involved in the major signaling pathways BCR/TCR, JAK/STAT and PI3K/AKT, as well as the involvement of the major heat shock protein families. CLL: chronic lymphocytic leukemia, MCL: mantle cell lymphoma, ABC-DLBCL: active B-cell diffuse large lymphoma, ALL: acute lymphoblastic leukemia, AML: acute myeloid leukemia, BL: Burkitt lymphoma, APL: acute promyelocytic leukemia, cHL: classical Hodgkin lymphoma, CML: chronic myeloid leukemia, MM: multiple myeloma MPN: myeloproliferative neoplasms.

The B-cell receptor (BCR) and T-cell receptor (TCR) are essential detectors of antigens on B and T cells, whose binding initiates multiple downstream signaling pathways leading to activation, survival, and differentiation. Their activations are also crucial in the development of leukemias and lymphomas, whose aberrant proliferation is often due to BCR pathway mutations [52]. Several HSPs have been involved in the proximal signaling of the BCR and TCR (Figure 1).

SRC refers to a family of proto-oncogenes encoding the lymphocyte-specific SRC family kinases (SFK). In this family, LCK (for lymphocyte-specific protein tyrosine kinase) is highly expressed by T-cell lymphoblastic leukemia (T-ALL) and is essential for TCR signaling [53,54]. Glucocorticoid resistance is reversed by LCK inhibition in pediatric T-ALL [55]. The inhibition of LCK by preventing its phosphorylation is an important strategy for the treatment of malignant hematopoiesis such as T-ALL, particularly with the use of bosutinib, dasatinib, or daracatinib, which affect the proliferation of leukemia cells [55,56,57]. Although it was shown several years ago that LCK interacts with HSP90 [58], only recently has the use of HSP90-specific inhibitors demonstrated the dependence of LCK expression on HSP90 in T-ALL [59]. The use of patient-derived models (PDX) has further shown the negative impact of HSP90 neutralization on primary leukemic cell survival [59].

LCK homologue protein LYN (Lck/Yes-related novel protein tyrosine kinase) is more specifically expressed by B-cell lymphoblastic leukemia (B-ALL) and B-cell lymphoma and is important for BCR signaling. The inhibition of LYN, notably with Dasatinib, is an important strategy for the treatment of B-ALL and B-cell lymphoma. LCK protects cells from glucocorticoid-induced apoptosis, and its inhibition enhances sensitivity to dexamethasone in lymphoma cells [60]. Lymphomas resistant to proteasome inhibitors show increased expression in BCR and activation of the BCR signaling pathway enhances the activity of SFK, especially LYN, and downstream kinases PI3K/AKT/mTOR in proteasome inhibitor-resistant lymphoma cells. Therefore, targeting BCR signaling with dasatinib could be a novel therapeutic approach for patients with mantle cell lymphoma that are refractory to proteasome inhibition with bortezomib. LYN is predominantly expressed in B-lymphocytes and plays a central role in initiating B-cell signaling. Evidence is mounting that strongly implicates an important role for LYN in several types of leukemia and lymphoma, particularly in B-ALL, where studies have confirmed the overexpression of LYN and its critical role in maintaining proliferation and antiapoptotic pathways in leukemic cells.

In CLL and MCL, high HSP90 expression correlates with overexpression of BCR signalosome proteins, including CD79a, PLCg2, LYN, BTK, and SYK. These proteins are associated with HSP90 in a multiprotein complex [21,61,62]. Pharmacological inhibition of HSP90 dislocates the complex and induces cell death, suggesting a critical role for maintenance of the tonic BCR signaling. Similarly, in ABC-DLBCL, HSP90 is a member of the BCR signalosome, and the HSP90 inhibitor PUH71 decreases SYK and BTK phosphorylation [63].

Considering ibrutinib resistance and given this BCR-complex, HSP90 targeting could be envisioned. Indeed, the HSP90 inhibitors SNX-5422 and AUY922 were recently shown to induce cell death in B cell lines expressing BTK C815S [62]. Combining drugs with ibrutinib to increase survival rates is being explored [64], and its association with PUH71 induced the synergistic killing of lymphoma cells [63]. In ABC-DLBCL, only 37% of patients respond to ibrutinib [65]. Phelan et al. have identified that most of these patients harbor dual CD79a and MyD88^L265P^ mutations leading to the formation of a cytoplasmic MyD88-TLR9-BCR supercomplex driving IκB phosphorylation and thereby to cell survival and proliferation [66]. We have shown that HSP110 expression correlates with MyD88 in patients’ lymph node biopsies and that HSP110, by chaperoning MyD88 and MyD88^L265P^, enhances ABC-DLBCL cell lines survival [24]. Therefore, specific inhibitors of HSP110, like the small chemical compound recently reported by us [67], would destabilize MyD88^L265P^ and subsequently the MyD88-TLR9-BCR super complex. This strategy might be a therapeutic alternative for ibrutinib-resistant patients.

In addition to chronic BCR-activated leukemia and lymphoma, HSP90 also cooperates with BCR signaling in Burkitt lymphoma (BL) as it interacts with SYK in a BCR Y197-dependent manner [68]. SYK/HSP90 interaction seems to play a particularly important role in BL cell survival, as LYN and BTK knock-down by shRNA do not alter the cells’ survival, unlike SYK knock-down.

BCR downstream pathways are subsequently impacted by HSP90 inhibition, as demonstrated in MCL, classical Hodgkin lymphoma (cHL), and ABC-DLBCL, where IkB degradation and reduction of NFkB signaling are observed [62,63,69]. In addition to indirect BCR effects, other client proteins of multiple signaling pathways are also impacted. In cHL, ERK/MAPK and RAS were reported to be down-regulated, and in ATL, AKT and the NfkB kinase complex IKKa, b, and d were degraded [70,71].

The involvement of HSP70 is less well documented than that of HSP90. However, in ALL it seems to play an important role. In this disease, the transforming growth factor-β-Activated Kinase 1 (TAK1), a member of the mitogen-activated protein kinase (MAP3K) family that has serine/threonine protein kinase activity, activates several pathways, including MAPK p38, JNK, and IKK [72]. HSP70 silencing leads to a decrease in the quantity of TAK1, which consequently derepresses Egr-1 expression, a tumor suppressor gene that leads to cell death [73]. Egr-1 low expression plays a major role in normal HSC long-term maintenance and localization; in contrast, its dysregulation is linked to hematopoietic aging [74]. This suggests that HSP70 would favor normal hematopoiesis through HSC regulation and the TAK1/Egr-1 pathway. Conversely, the Egr-1 down-expression observed in AML, ALL, and CML could be reversed by targeting HSP70 [75].

### 3.2. Chaperoning the JAK/STAT Pathway

Aberrant activation of the Janus-activated kinase (JAK) tyrosine kinase family plays an oncogenic role in leukemias, lymphomas, and other hematopoietic disorders such as myeloproliferative neoplasms (MPN). TYK2 was the first identified JAK family member aberrantly activated in T-ALL and B-ALL. It results in STAT1 activation and up-regulation of the anti-apoptotic protein BCL2 [76,77]. TYK2 has been identified as a HSP90 client protein [58], and in T-ALL, pharmacological inhibition of HSP90 led to TYK2 degradation, reduced STAT1 phosphorylation, and subsequent BCL2 down-expression [78].

In MPN, the JAK2 mutation has been observed in most patients, ranging from 50% with essential thrombocythemia and myelofibrosis to 95% in polycythemia vera [79]. Therefore, JAK inhibitors like ruxolitinib are being used in the clinic. In patients, JAK2 inhibition has been shown to decrease splenomegaly but cannot eradicate the MPN clone [80], leading to the search for alternative targeted therapies. Of note, JAK2 is a client protein of HSP90, and its inhibition leads to JAK2 degradation, followed by abrogation of the JAK/STAT signaling in vitro and in vivo [81,82]. Murine models of MPN treated with a HSP90 inhibitor led to global improvements in the mice with significant survival benefits. This prompted a clinical pilot study with HSP90 inhibitors [83], which showed an in vivo reduction in the spleen’s size in all patients. Although these results need to be confirmed in a more ambitious clinical trial, they suggest that HSP90 targeting would be a valuable therapy in MPN. An additional interest in HSP90 inhibitors would be in patients resistant to JAK inhibitors due to JAK2 mutations (G935R, Y931C, and E864K), because those mutant JAK2 are still client proteins of HSP90 [84].

Concerning small HSPs, HSP27 also plays a role in the myelofibrosis form of MPN as it is strongly expressed in bone marrow biopsies of patients in contrast to HSP70 and HSP90 [85]. HSP27, indeed, binds directly to JAK2 and STAT5 to protect STAT5 from its dephosphorylation. It might also favor the molecular assembly JAK2/STAT5. Furthermore, treatment of murine models of myelofibrosis with OGX-427, an antisense oligonucleotide against HSP27, limited the progression of bone marrow fibrosis, normalized the platelet and white blood cell counts.

In cHL, JAK2 and TYK2 activation are also observed and correlate with STAT signaling and cell survival [86]. As in MPN, HSP90 inhibition in cHL and multiple myeloma (MM) leads to loss of STAT3 and 5 tyrosine phosphorylation due to JAK1,3 and TYK2 down-regulation [86,87]. Receptor expression involved in the JAK/STAT3 pathway activation, such as IL6-R and IGRI-R, are also decreased upon HSP90 inhibition in MM [88]. In conclusion, several cancers with JAK2/STAT overactivation, either induced by growth factor stimulation or by receptor mutations, could benefit from HSP90 or HSP27 targeting alone or in combination with JAK2 targeting therapies.

### 3.3. Chaperoning the PI3K/AKT Pathway

The phosphatidylinositol 3-kinase (PI3K)/AKT regulates many cellular processes that favor cell survival and proliferation in normal and pathological hematopoietic cells. The hyperactivation of this pathway is frequently observed in leukemia and lymphomas due to either mutations or gene amplifications of PI3K or AKT, but also due to the loss of regulatory proteins such as PTEN [89]. Given the importance of this pathway, many inhibitory compounds have been developed for clinical use and are now being tested [90]. HSP90 and HSP70 have been shown to regulate this pathway.

HSP90 not only binds to AKT with CDC37 in an active complex to promote AKT activity and PI3K signaling [91,92], but also binds to multiple components of the PI3K/AKT/mTOR pathway (the PI3K catalytic and regulatory subunits, as well as downstream factors mTOR and EIF4E), as shown in BL [93]. As mentioned previously for the association of other signaling pathway inhibitors with HSP90 inhibitors, a synergistic anti-tumor effect was observed with PU-H71 and PI3K/mTOR inhibitors in BL [93]. In AML, the combination of ganetespib with cytarabine also provides synergistic cytotoxicity and is characterized by AKT disappearance [94]. For GC-DLBCL that shows a great dependency on AKT mediated by SYK activation [95,96], HSP90 targeting seems a good option to neutralize two client proteins essential for lymphoma survival. This goal is, however, reversed in acute promyelocytic leukemia (APL), which lacks AKT activation and shows a reduction in HSP90 expression. In this situation, ATRA treatment reverses HSP90 expression and AKT phosphorylation, suggesting a role for HSP90 in restarting myeloid differentiation. Regarding HSP70, the HSP70 inhibitor PFT-μ has been shown to reduce AKT expression in ALL and AML, also suggesting a chaperoning role in this pathway [97]. However, solid demonstrations of direct interaction and regulation are still lacking in those pathologies.

## 4. HSPs Are Chaperones of Oncogenes

**Figure 2:**
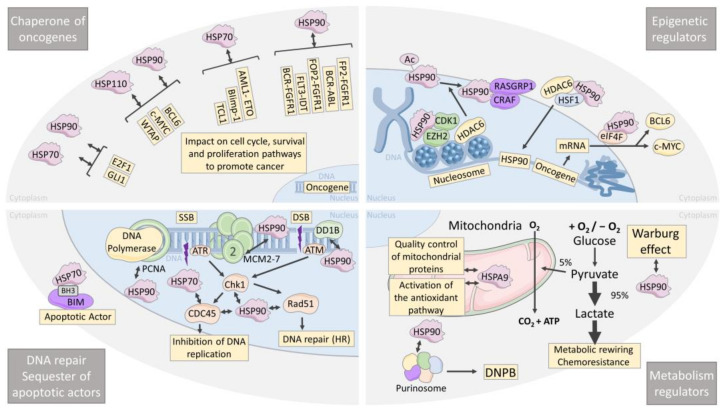
HSP are chaperones of oncogenes, epigenetic regulators, metabolism regulators, chaperones of DNA repair, and sequesters of apoptotic actors in leukemia and lymphoma. Schematic of a leukemia/lymphoma cell showing the four major roles of HSPs in the global cellular homeostasis, which are: chaperones of oncogenes, chaperones of epigenetic regulators, chaperones of DNA repair actors, and their involvement in metabolism. SSB: single-strand break, DSB: double-strand break, DNPB: de novo purine biosynthetic pathway, HR: homologous recombination.

Fusion proteins play a critical role in many leukemia initiations, such as the well-known BCR-ABL fusion in CML. As they are not naturally synthesized and folded proteins, HSPs are mandatory to maintain their correct conformation (Figure 2). BCR-ABL is a client protein for HSP90. The chaperone interacts with its coiled-coil domain in the N-terminal part, thereby preventing the transport of BCR-ABL to the nucleus and permitting the cytosolic promotion of signaling [98]. Conversely, HSP90 inhibition leads to the nuclear transport of BCR-ABL, its degradation, and a subsequent decrease of the downstream signaling molecules P-AKT and P-STAT5 [98,99]. Recently, a newly identified HSP90-specific inhibitor that targets HSP90 dimerization sites, aminoxyrone, has shown a similar effect on BCR-ABL down-expression in both leukemic stem cells and bulk fraction [100]. Other fusion proteins like FOP2-FGFR1, FLT3-ITD, and BCR-FGFR1 in AML are also client proteins of HSP90/CDC37, which holds them in a permanently active conformation [101,102,103]. HSP90 inhibitors alone or in combination with cytarabine show FP2-FGFR1 downregulation and anti-leukemic activity [104]. Similarly, the association of HSP90 inhibitors with a protein translation inhibitor (homoharringtonine) shows synergistic apoptosis and cell cycle arrest effects in FLT3-ITD AML [105]. Furthermore, this strategy is still valid in patients previously treated in the long term with TK inhibitors for FLT3-ITD and developing resistance through acquired mutations in FLT3-ITD, as HSP90 inhibitors are still very effective in those mutated cells [106]. Cooperation between different HSP family members is sometimes necessary to achieve the correct folding of aberrant proteins. This is the case for the AML1-ETO fusion proteins, the most important oncogenes in AML, whose correct folding is enabled by the associated work of both HSP70 and the chaperonin TRiC [107].

Aberrant transcriptional regulation by either hyperactive transcription factors or transcriptional repressors is also a hallmark of leukemia/lymphoma cells in which HSPs are involved. Bcl6, the transcriptional suppressor highly expressed in GC-DLBCL, and c-MYC, the master cell cycle regulator that promotes BL development, are both sustained by HSP90 and HSP110 (Figure 2). Indeed, HSP90 was shown to bind to BCL6 at the promotor of the targeted genes, stabilizing BCL6 and facilitating BCL6 transcriptional activity [108]. HSP110 expression correlates with c-MYC and Bcl6 protein expression in tumor biopsies of BL, DLBCL, FL, and MCL, and HSP110 knockdown leads to decreases in both oncogenes [23]. As described for the contribution of HSP90 to various signaling pathways, HSP90 is also embedded in multiprotein complexes in the nucleus as it is associated with BCL6 and WTAP (Wil’s tumor-1-associated protein) [109]. C-MYC is also a HSP90 client protein in BL and MCL [110,111], and therefore its degradation by the proteasome is protected by the chaperone.

Although BCL6 and c-MYC are promising therapeutic targets, effective inhibitors have not yet reached clinical efficacy, suggesting that indirect inhibition through the destabilization of their protein complexes with HSPs could be an alternative strategy. This is supported by reports of the degradation of BCL6 and c-MYC upon HSP90 or HSP110 inhibition, which is followed by increased cell death. Of note, in MCL, the MYC transcriptional program was particularly and specifically suppressed upon HSP90 inhibition compared to other pathways, confirming the importance of the peculiar HSP90/c-MYC relationship [110]. Many other transcription factors might be regulated by HSP70, HSP90, and HSP110, and future research will probably confirm this. For instance, E2F1, a central factor involved in cell cycle in MCL, and GLI1, the main effector of the hedgehog pathway in AML, have been identified as new HSP90 client proteins that are down-expressed upon HSP90 and HSP70 inhibition [112,113]. Conversely, the accelerated cytosolic degradation of the mutated transcription factors that would, in their wild-type form, normally alleviate the aborted differentiation is a not-so-well-studied mechanism of aberrant HSP chaperoning. This has been demonstrated in ABC-DLBCL, where unstable N-terminally misfolded Blimp-1 mutants are recognized by HSP70 and brought to Hrd1-mediated cytosolic sequestration, ubiquitination, and proteasomal degradation. In this context, HSP70 inhibition restored Blimp-1 mutants’ nuclear localization and function [114]. This mechanism of HSP70-mediated aberrant sequestration of a transcription factor is reminiscent of what we observed for GATA-1 in myelodysplastic syndromes [115].

T-cell leukemia/lymphoma 1 (Tcl1) is overexpressed in aggressive CLL and other human B-cell lymphomas. It is known to be a co-activator of AKT and a transactivator of NFkB [116,117]. HSP70 inhibition induces the proteasomal degradation of TCL1, prevents NFkB activation, and decreases tumor growth in mice xenografted with Daudi and Raji BL cell lines [118]. Therefore, not only transcription factors but also oncogenes that serve as signaling pathway transactivators, such as Tcl1, are stabilized by HSPs, expanding the number of putative therapeutic targets (Figure 2).

## 5. HSPs Are Epigenetic Regulators

Epigenetic regulators are also masters of gene expression, and their down- or over-expression is frequently associated with chemotherapy resistance [119]. In particular, the histone methyltransferase EZH2 is lost in 50% of AML patients and induces cytarabine resistance [120]. This reduction in EZH2 protein is due to CDK1-dependent phosphorylation within a complex involving HSP90. Accordingly, HSP90 inhibition restores EZH2 and drug sensitivity [121]. The histone deacetylase (HDAC) proteins are powerful epigenetic translational regulators. They mediate chromatin compacting and gene expression by the deacetylation of histones and regulate both the protein stability and activity of non-histone proteins. HDAC6 is a client protein of HSP90 and mediates its deacetylation [122,123]. Therefore, HDAC inhibition leads to HSP90 hyperacetylation and degradation, with all the cascade of client protein disappearance that follows (Figure 2). Particularly, in B-cell lymphomas and T-cell leukemias, RASGRP1 and CRAF, two HSP90 client proteins, are degraded upon HDAC6 inhibition, leading to RASGRP1/CRAF-dependent apoptosis [124]. Being an HSP90 client protein, however, HDAC6 is also degraded upon HSP90 inhibition; therefore, mutual regulation does exist and is a tempting target for combinational therapies. Furthermore, in AML and CML, HDAC inhibition leads to stronger binding of HSP90 to its inhibitor, 17-allylamino-17-demethoxygeldanamycin (17-AAG), fostering HSP90 degradation. Accordingly, combinations of HDAC inhibitors and 17-AAG logically result in increased cell death [125]. Finally, the transcriptional regulation of HSP90 is also modulated by HDAC6, first by facilitating HSF1 nuclear translocation, and second by deacetylating histone at the HSP90 gene locus. In conclusion, the search for dual HDAC and HSP90 inhibitors is a promising field to synergistically improve therapeutic strategies [126]. Transcriptional regulation of many oncogenic mRNAs is also controlled by the eukaryotic initiation complex eIF4F, which binds to HSP90 [127]. As in DLBCL, BCL6 and cMYC mRNAs are exported and translated under the control of eIF4F; targeting HSP90 would also make it possible to blunt the production of these oncogenes. In conclusion, HSP90 is known to be a direct regulator of gene expression as a chromatin-bound protein [128].

## 6. HSPs Are Leukemia and Lymphoma Metabolism Regulators

Metabolism rewiring allows tumor cells to adapt their biosynthesis to the nutrient availability in their microenvironment. Therefore, aggressive lymphomas and leukemias both use oxidative phosphorylation and glycolysis to fulfill their need in ATP. The latter process is amplified and known as the Warburg effect, which contributes to chemoresistance [129]. Oncogenes and aberrant cell signaling such as c-MYC, HIF1a, and the PI3K/AKT/mTOR pathway contribute to the promotion of the Warburg effect, the conversion of pyruvate to lactate, and fatty acid synthesis. By sustaining these pathways and oncogenes directly or indirectly, HSPs can modulate such metabolic rewiring (Figure 2). Recently, it has been reported that HSP90 is involved in this process as a stabilizer of protein complexes rather than as signaling pathway promoters [130]. Indeed, HSP90 coordinates and sustains several metabolic pathways, such as enzymes involved in the metabolism of nucleotides, carbohydrates, and amino acids, by promoting the formation of multienzymatic complexes. More recently, this metabolic function of HSP90 has also been shown in the process of de novo purine biosynthesis (DNPB) under high purine demands during metabolic stress [131]. Purinosome, a multienzymatic complex that drives DNPB, requires HSP90 to maintain its physical properties and thereby promote purine production. Although demonstrated in HeLa cells and not yet in hematological malignancies, this finding supports the role of HSP90 as a metabolic regulator and encourages future research in this field.

Mitochondria are multicompetent actors in cellular metabolism, and mitochondrial chaperones are important factors. HSPA9 (Mortalin), a mitochondrial HSP70 family member, is involved in the quality control of proteins imported into the mitochondrial matrix. HSPA9 also senses oxidative stress and controls the activation of the antioxidant pathway [132], therefore playing a central role in the mitochondrial stress response. The targeting of mitochondrial chaperones could be envisioned as sensitivity to proteostasis inhibition strategies, commonly used in relapsed MM or MCL patients through bortezomib treatment, which relies on mitochondrial metabolism [133]. This hypothesis is sustained by the fact that inhibition of HSPA9 by HSP70 allosteric inhibitors alleviates proteasome inhibitor resistance in MM cells [134,135]. Globally, HSP70 family members are also good target candidates, as resistance to Bortezomib therapy is associated with their up-regulation [135,136].

## 7. HSP Are Chaperones of DNA Repair and Sequesters of Apoptotic Actors

DNA damage can result from environmental or endogenous stress, consequently initiating several mechanisms of DNA repair to maintain cells’ integrity and survival. The ability of HSPs to promote DNA repair in several cancers has been recently reviewed [137,138]. Here, we will focus on their role in leukemia and lymphomas (Figure 2). Several mechanisms are involved in DNA repair, i.e., DNA damage detection, cell cycle arrest, and DNA synthesis [139]. PCNA (proliferating cell nuclear antigen), which favors the recruitment of DNA polymerase to DNA strand breaks, DDB1 (damage-specific DNA binding protein), which recognizes UV-induced DNA lesions, and MC2, a substrate of ATM and ATR upon DNA damage, are reduced upon HSP90 inhibition by SNX-7081 in CLL [140]. This promotion of DNA repair by HSP90 favors resistance to DNA-damaging drugs like fludarabine. Therefore, treatment with an HSP90 inhibitor restores the cells’ sensitivity [141,142]. Similarly, in AML, whose treatment relies mostly on replicative stress induced by nucleoside analogues, HSP90 inhibition reduces Chk1 and Rad51, two HSP90 client proteins that induce cell cycle arrest and promote homologous reparation repair (HR), respectively. Accordingly, treatment of HSP90 inhibitors with cytarabine leads to a stronger cell cycle arrest [143]. Therefore, associating an HSP90 inhibitor with nucleoside analogues for the treatment of AML would permit increasing leukemic cells’ responses and limiting the progression of the disease. Regulation of DNA replication and repair could also be modulated at the transcriptional level by E2F1, a client protein of HSP90 and HSP70 that assures the transcription of CDC6, CDC45, MCM4, MCM7, RIM1, and RIM2 in CLL [112,113]. The subtle equilibrium between pro- and anti-apoptotic proteins must be finely tuned. HSP are involved in this adjustment and could shift the balance toward cell survival in cancer cells through direct interaction with key apoptotic proteins. In hematopoietic cells, interaction of HSP70 with the BH3-only pro-apoptotic protein BIM via a BH3 domain has been shown [144]. A dual mechanism simultaneously appears; first, sequestration of BIM from Bcl2, which contributes to cell survival, and second, promotion of the chaperone activity of HSP70 with the aid of BIM, which serves as a co-chaperone. Recently, a specific inhibitor that targets the BH3 domain involved in HSP70-BIM interaction has been shown to disrupt this association and thereby overcome the BCR-ABL independent TKI resistance in CML [145]. Venetoclax is a Bcl2-selective inhibitor approved in 2016 as a treatment of CLL, AML, and shows promising results in MM. Recent studies have shown the efficacy of this inhibitor as a treatment for MCL patients refractory to ibrutinib [146]. However, the development of acquired resistance in some patients remains inevitable. It has been shown that HSP27 may be the cause of this relapse in patients treated with venetoclax for MCL, suggesting that targeting HSP27 could overcome this resistance [147]. Furthermore, in MCL, a robust synergy was observed between the HSP90 inhibitor, LAM-003 and venetoclax in FLT3-ITD AML cells, a particularly aggressive and resistant cell type [148].

## 8. Recent Progress in Clinical Targeting of HSP90

As said previously, HSP90 is known to facilitate the maturation, stabilization, and activation of over 200 client proteins, covering all cellular processes in cancers. Therefore, huge efforts have been made over the past decades to develop selective inhibitors that directly target HSP90. Most of the HSP90 inhibitors that are currently available and all that have been clinically assessed both in solid and hematological tumors, bind to the nucleotide binding pocket of the N-terminal domain and block the processing of client proteins by preventing ATP binding and hydrolysis.

HSP90 inhibition has shown, for example, some efficacy for the treatment of lymphomas. HSP90 inhibitor ganetespib enhances the sensitivity of MCL to Bruton’s Tyrosine Kinase inhibitor ibrutinib [149]. Aberrant HSP90 expression in lymphocytes and HSP90 response to anti-PD-1 therapy in lymphoma patients have also been shown [150]. HSP90 inhibition sensitizes diffuse large B-cell lymphoma cells to Cisplatin [151]. Regarding AML, several HSP90 inhibitors affect cancer cells’ growth. For instance, HSP90 inhibitors overcome the resistance to Fms-like tyrosine kinase 3 (FLT3) inhibitors in AML [106]. HSP90 inhibitors NVP-AUY922 and ganetespib (STA-9090) have shown synergistic anti-leukemic activity with cytarabine in AML [94,104]. Alvespimycin (17-DMAG), administered intravenously twice weekly to AML patients, was also found to be effective [152]. Co-treatments with 17-AAG and a FLT3 kinase inhibitor or a histone deacetylase inhibitor are highly effective against human AML cells with mutant FLT3 [153,154]. Finally, HSP90 inhibition depletes DNA repair proteins to sensitize AML to nucleoside analog chemotherapeutics [143].

Other studies have also confirmed that there is an elevated expression of HSP90 in CML, suggesting that HSP90 could serve as a prognostic marker [20]. This also explains why several chemical inhibitors of HSP90 have been tested to treat CML [155]. In addition, targeting HSP90 dimerization is effective in imatinib-resistant CML [100]. ACY-1215 suppresses the proliferation and induces the apoptosis of CML cells via the ROS/PTEN/Akt pathway [156]. Preclinical evaluation of the HSP90 inhibitor SNX-5422 in Ibrutinib-resistant CLL has furthermore been described, and destabilization of ROR1 enhances the activity of Ibrutinib against CLL in vivo [64,157].

Regarding ALL, the HSP90 inhibitor PU-H71 has also been shown to be effective in treating T-ALL patient samples that express a high level of NOTCH1 (notch receptor 1) [158]. TAS-116 (pimitespib), a HSP90 inhibitor, shows efficacy in preclinical models of adult T-ALL [159].

It is well known that HSP90 is involved in many essential cellular processes in non-malignant cells, such as protein maturation and stabilization, chaperoning of kinases, transcription factors, and other major signaling proteins [160,161]. Fortunately, tumor cells rely more on HSP for survival and proliferation than normal cells, limiting the inhibitors’ side effects [161]. However, to limit these adverse events, one must pay attention to each HSP90 inhibitor’s selectivity towards normal cells, as AUY922 does not preferentially impair the proliferation of cancer cells over normal cells, whereas the C-terminal Hsp90 modulator SM258 only impacts colon cancer cell lines [162]. In monotherapies, HSP90 inhibitors have shown some toxicity, limiting their efficacy, and tumor cells manage to develop resistance [161]. Therefore, combining low doses of HSP90 inhibitors with other anti-cancer drugs would be a solution in the future. Development of inhibitors that would target the tumor-specific conformation of HSP90 would also be a goal [162,163]. Current HSP inhibitors and their stage of clinical development are shown in Table 1.

## 9. Discussion

Leukemia and lymphoma represent a diverse, although particular, group of cancers that poses specific challenges to researchers. We have presented here the diverse and major roles of HSPs in these diseases, which could help to better target oncogenic processes and contribute to improving therapies. Typical of B- and T-cell populations, BCR and TCR signaling networks are frequently and heavily mutated in lymphoid leukemias and lymphomas and strongly rely on HSPs to transmit sustained signaling. We have also described the stabilization of many oncogenes by HSPs that would otherwise endanger the viability of tumor cells.

We must not forget that the roles of HSP and HSF are not limited to cell signaling but provide critical support to gene expression and epigenetic regulation, particularly through HDAC6. In solid tumors, HSF1, the major HSP transcription factor, also controls various transcriptional programs such as those for cell-cycle regulation, RNA splicing, and resistance to apoptosis [164]. Very few studies have explored these non-classical functions of HSF1 in hematological malignancies. We have shown that HSF1 is highly expressed during myeloid differentiation, modulating PU.1 expression [165]. Of note, chronic myelomonocytic leukemia cells that are frequently associated with defective monocyte differentiation were lacking both HSF1 and PU1 expression [165]. HSF1 is also known to be involved in metabolic regulation in hepatocellular carcinoma [166]. This could be explored in the context of leukemia and lymphomas as metabolic rewiring is modulated by HSP90 and HSPA9, promoting the production of large amounts of needed ATP [130]. Targeting HSF1 has been difficult to date due to the lack of specific inhibitors. However, recently, Dong et al. [167] identified a small molecule that directly binds to HSF1 and promotes the degradation of its nuclear and active forms. This promising new tool should soon be tested in leukemias and in lymphomas murine models to validate its clinical perspectives.

So far, most studies on the role of HSPs have focused on HSP90 and HSP70 for obvious reasons of cellular abundance and inhibitor availability. This must not overshadow the importance of other HSPs such as HSP27, mitochondrial HSPA9, or HSP110. The latter starts to reveal its critical involvement in the oncogenesis of lymphoma, such as the stabilization of several oncogenes like BCL6, C-MYC, and MyD88. The recent discovery of the first HSP110 inhibitors [67] would probably foster research in other types of hematological malignancies and the identification of more functions.

In this review, we focused on the intracellular functions of HSPs, but one must not forget the extracellular HSPs, either membrane-bound or secreted in the extracellular milieu. HSP70 and HSP110 are expressed on the surface of NHL or released within extracellular vesicles such as exosomes and have the ability to interact with various ligands expressed on immune cells like TLR2 or TLR4 [22,168,169]. These extracellular HSPs are mainly observed in tumors, both in cells and their microenvironment, and have the capacity to activate immune cells associated with immunosuppression [170,171,172]. Therefore, their targeting or neutralization should be envisioned. This could be achieved by CAR-T cell technology as proposed by Smith J. et al. for HSP70 (patent: EP3250605).

In B cell lymphomas, exosomes from plasma samples can be easily isolated and characterized, and their content might be used as a predictor of response to therapy [173]. Moreover, HSP27, HSP70 and HSP110 exosomes can be easily detected in body fluids, and their content is associated with a bad prognosis in several solid tumors [85,174,175,176]. Therefore, their detection in plasma samples from patients with leukemia and lymphoma may also be used as biomarkers to predict tumoral HSP expression and to monitor responses to therapies.

## 10. Conclusions

In conclusion, HSPs appears to play multiple and crucial roles in every aspect of leukemia and lymphoma biology, from specific cell signalling to epigenetic regulations. Identification of specific functions related to the oncogenesis of these cells must be pursued to provide better targeted and personalized therapies. Identification of new and more specific inhibitors will also be beneficial for the entire anti-cancer field of research as HSPs are hallmarks of cancer resistance to intra- and extracellular stresses.

## Figures and Tables

**Table 1:** List of HSP inhibitors presented in this review, showing their targets, mechanisms of action, and clinical phases of development.

HSP Inhibitors	Target	Mechanism of Action	Phase of Development	Hematological Malignancies
Foldamers 33 and 52	HSP110	Binding to the ATP binding domain	Pre-clinical research	ABC DLBCL [67]
PUH71 (Zelavespib)	HSP90	Binding to the ATP binding domain	Clinical: Phase I	NHL NCT01269593/MPN NCT01393509
			Pre-clinical research (with Ibrutinib)	ALL/ABC DLBCL [63]
			Pre-clinical research (with PI3K/mTOR inhibitors)	BL [93]
SNX-5422 (PF-04929113)	HSP90	Convert to SNX-2112 and binding to the ATP binding domain	Clinical: Phase I (with Ibrutinib)	CLL [64,157]
SNX-7081	HSP90	Binding to the ATP binding domain	Pre-clinical research (with Fludarabine)	CLL [140]
NVP-AUY922 (Luminespib)	HSP90	Binding to the ATP binding domain	Pre-clinical research (with Cytarabine)	AML [94,104]
Aminoxyrone	HSP90	Binding to C-terminal dimerization domain	Pre-clinical research	CML [100]
17-AAG (Tanespimycin)	HSP90	Binding to the ATP binding domain	Clinical: Phase II	Hodgkin/MCL/ALCL/MM (NCT00117988)
			Pre-clinical research (with HDAC inhibitors)	AML/CML [125]
LAM-003 (MPC-3100)	HSP90	Convert to LAM-003A	Clinical: Phase I	AML [148]
Ganetespib	HSP90	Binding to the ATP binding domain	Clinical: Phase I	MM (NCT01485835)
			Pre-clinical research (with Ibrutinib)	MCL [149]
			Pre-clinical research (with cytarabine)	CML [94]
17-DMAG (Alvespimycin)	HSP90	Binding to the ATP binding domain	Clinical: Phase I	Lymphoma & leukemia (NCT00089271)
			Pre-clinical research	AML CLL [71]
TAS-116 (Pimitespib)	HSP90	Binding to the N-terminal domain of HSP90	Pre-clinical research	ALL [159]
PFT-µ (Pifithrin-μ)	HSP70	Binding to the C-terminal domain of HSP70	Pre-clinical research	ALL/AML [97]
VER-155008	HSP70	Binding to the ATP binding domain	Pre-clinical research	MM [135]
JG-98	HSP70	Allosteric HSP70 inhibitors	Pre-clinical research	MM [134]
DTHIB	HSF1	Binding to the DNA binding domain	Pre-clinical research	AML [31]

AML: acute myeloid leukemia; ALL: acute lymphocytic leukemia; MM: multiple myeloma; CML: chronic myeloid leukemia; MCL: mantle cell lymphoma; ALCL: anaplasic large cell lymphoma; CLL: chronic lymphoblastic leukemia; MPN: myeloproliferative neoplasms; ABC-DLBCL: activated B cell diffuse large B cell lymphoma; NHL: non-Hodgkin lymphoma; BL: Burkitt lymphoma.
